# Loneliness, physical activity, and mental health during COVID-19: a longitudinal analysis of depression and anxiety in adults over the age of 50 between 2015 and 2020

**DOI:** 10.1017/S1041610220004135

**Published:** 2020-12-17

**Authors:** Byron Creese, Zunera Khan, William Henley, Siobhan O’Dwyer, Anne Corbett, Miguel Vasconcelos Da Silva, Kathryn Mills, Natalie Wright, Ingelin Testad, Dag Aarsland, Clive Ballard

**Affiliations:** 1 University of Exeter Medical School, College of Medicine and Health, Exeter, UK; 2 Department of Old Age Psychiatry, Institute of Psychiatry, Psychology and Neuroscience, King’s College London, London, UK; 3 Global Public Health, Public Health England, London, UK; 4 Centre for Age-related Medicine – SESAM, Stavanger University Hospital, Stavanger, Norway

**Keywords:** COVID-19, mental health, loneliness, physical activity, exercise, depression, anxiety, pandemic

## Abstract

**Objective:**

Loneliness and physical activity are important targets for research into the impact of COVID-19 because they have established links with mental health, could be exacerbated by social distancing policies, and are potentially modifiable. In this study, we aimed to identify whether loneliness and physical activity were associated with worse mental health during a period of mandatory social distancing in the UK.

**Design:**

Population-based observational cohort study.

**Setting:**

Mental health data collected online during COVID-19 from an existing sample of adults aged 50 and over taking part in a longitudinal study of aging. All had comparable annual data collected between 2015 and 2019.

**Participants:**

Three-thousand two-hundred and eighty-one participants aged 50 and over.

**Measurements:**

Trajectories of depression (measured by PHQ-9) and anxiety (measured by GAD-7) between 2015 and 2020 were analyzed with respect to loneliness, physical activity levels, and a number of socioeconomic and demographic characteristics using zero-inflated negative binomial regression.

**Results:**

In 2020, PHQ-9 score for loneliness, adjusted for covariates, was 3.23 (95% CI: 3.01–3.44), an increase of around 1 point on all previous years in this group and 2 points higher than people not rated lonely, whose score did not change in 2020 (1.22, 95% CI: 1.12–1.32). PHQ-9 was 2.60 (95% CI: 2.43–2.78) in people with decreased physical activity, an increase of .5 on previous years. In contrast, PHQ-9 in 2020 for people whose physical activity had not decreased was 1.66, 95% CI: 1.56−1.75, similar to previous years. A similar relationship was observed for GAD-7 though the absolute burden of symptoms lower.

**Conclusion:**

After accounting for pre-COVID-19 trends, we show that experiencing loneliness and decreased physical activity are risk factors for worsening mental health during the pandemic. Our findings highlight the need to examine policies which target these potentially modifiable risk factors.

## Introduction

In order to contain and reduce the spread of COVID-19, the UK government introduced nationwide lockdown measures on March 23, 2020 which restricted time permitted outside and all nonessential in-person contact. Those with certain high-risk medical conditions were advised to “shield” (i.e. not leave the house for 12 weeks) and those aged 70 and over were advised to strictly adhere to the restrictions. The potential mental health impacts of this type of policy have been highlighted in a number of high-profile commentaries, with possible mechanisms including the pressures of lockdown, anxieties about infection and the knock-on economic consequences (Armitage and Nellums, 2020; Duan and Zhu, 2020; Galea et al., 2020; Gunnell *et al.*, 2020; Holmes *et al.*, 2020; Hwang *et al.*, 2020; Jeste, 2020; Pfefferbaum and North, 2020; Yao *et al.*, 2020). Previous research into mental health in the pandemic has largely focused on socioeconomic, demographic, and clinical comorbidities, with younger age, female gender, and low socioeconomic status being consistently associated with higher risk (COVID-19 Psychological Research Consortium (C19PRC), 2020; Frank *et al.*, 2020; McGinty *et al.*, 2020; Pierce *et al.*, 2020). While these links are undoubtedly important, research must also focus on potentially modifiable risk factors.

Loneliness and physical activity are critical mediators of mental health, and therefore warrant close consideration during the pandemic (Armitage and Nellums, 2020; Haskell *et al.*, 2009; Santini *et al.*, 2020). The pandemic may lead to low activity levels and exacerbate the relationship between loneliness and mental health in some (e.g. through social distancing and movement restrictions) and as such, they may represent modifiable targets for resilience and management programs (Treichler *et al.*, 2020). Specifically, there is evidence from other contexts that both loneliness and physical activity can be modified (Fakoya et al., 2020; García-Hermoso *et al.*, 2020). The first step is understanding what links exist between loneliness, physical activity, and mental health during the pandemic. Longitudinal data covering the pre-pandemic and pandemic period is needed to address this key question. Three representatively sampled surveys (two USA and one UK) with data pre- and during the pandemic reported no significant changes in loneliness, but the interactions with the onset of the pandemic on mental health levels were not examined (Luchetti *et al.*, 2020; McGinty *et al.*, 2020; Office for National Statistics, 2020). Two cross-sectional studies have linked loneliness with worse mental health and psychological distress, and a third indicated that people with low social support (a possible proxy for loneliness) had a more severe trajectory of depression during the pandemic (Frank *et al.*, 2020; Frenkel-Yosef *et al.*, 2020; Killgore *et al.*, 2020). However without data prior to 2020, it is impossible to evaluate fully the specific importance of these factors during the pandemic. In particular, it will be important to understand whether these relationships reflect well-established links between loneliness and mental health or whether there was a specific effect of the pandemic. Though highlighted as important in commentaries, there has been little research into the links between physical activity and mental health; to our knowledge, the only published study used a cross-sectional design (Maugeri *et al.*, 2020).

To address the gap in research around the impact of loneliness and physical activity on mental health during COVID-19, we analyzed data from 3281 participants, all of whom had mental health data available from before the pandemic. We hypothesized that trajectories of depressive and anxiety symptoms in people who were lonely or whose physical activity had decreased during the pandemic would be adversely affected. In addition, we also examined a number of other demographic and socioeconomic variables on mental health trajectories.

## Method

### Study design and setting

The study was conducted with participants from the PROTECT study. PROTECT is a longitudinal study of mental and cognitive health, with annual assessment, in people over the age of 50 on enrollment which was launched in November 2015 (http://www.protectstudy.org.uk/) (Creese *et al.*, 2019). In April 2020, there were 24,030 people enrolled in PROTECT. Written informed consent was obtained online from all participants.

On May 13, 2020, around 4.5 years after PROTECT started, a specific COVID-19 mental health questionnaire was launched in PROTECT, again completed online. All 24,030 participants were invited by email to complete the COVID-19 questionnaire and taking part was voluntary. Here, we present an analysis of data collected between May 13 and June 8, 2020, combined with existing data from previous years.

The authors assert that all procedures contributing to this work comply with the ethical standards of the relevant national and institutional committees on human experimentation and with the Helsinki Declaration of 1975, as revised in 2008. All procedures involving human subjects/patients were approved by the UK London Bridge National Research Ethics Committee (Ref: 13/LO/1578) and the COVID-19 mental health questionnaire was approved by the same committee (as an amendment) on April 6, 2020.

### Participants

The PROTECT cohort includes people aged 50 or over at enrollment living in the UK. Additional inclusion criteria are access to a computer and internet, able to read and write English, and no diagnosis of dementia. All participants who opted in to receive study communications were invited to complete the COVID-19 mental health questionnaire.

### Variables

The principal outcome measures for this study were PHQ-9 and GAD-7 scores (measuring depression and anxiety, respectively). These were recorded pre-pandemic (2015–2019) as part of the main PROTECT study, and during the pandemic (2020) as part of the COVID-19 mental health questionnaire.

### PROTECT pre-pandemic data collection 2015–2019

Before the pandemic, all participants completed a series of online self-report questionnaires, which included demographic information (date of birth [in this study, age in 2020 was used], gender, highest level of education [left school at 16, left school at 18, undergraduate degree, postgraduate degree], employment status [full-time, part-time, self-employed, retired, unemployed], marital status [married/civil partnership/cohabiting, widowed/divorced/separated, single], and history of psychiatric and physical illness). In addition, mental health assessments by PHQ-9 and GAD-7 were completed annually prior to the pandemic.

Depression was assessed with the PHQ-9, a 9-item questionnaire, which assesses the frequency of depressive symptoms over a 2-week window (Kroenke *et al.*, 2001). Each item is rated on a 4-point scale (0 = not at all; 1 = several days; 2 = more than half the days; 3 = nearly every day) and a total score (maximum 27) is obtained by adding the nine items. Anxiety was assessed with the GAD-7, a 7-item questionnaire assessing the frequency of anxiety symptoms over a 2-week window (Spitzer *et al.*, 2006). The ratings are the same as PHQ-9, with the maximum total score being 21 (7 × 3). For both scales, a threshold of 5 or above on the total score is indicative of mild symptoms and 10 or above is indicative of moderate or severe symptoms.

Participants completed up to four annual GAD-7 and PHQ-9 assessments spread over 5 years between 2015 and 2019 (depending on enrollment date). Enrollment to PROTECT is open continuously and started with a national publicity drive in October and November 2015, and as a result, the majority of current participants enrolled in those 2 months. For those who completed the COVID-19 mental health questionnaire, this figure was 1930 (59%). After the initial wave of enrollment, 405, 382, 338, and 18 enrolled in 2016, and in 2017, 2018, and 2019, respectively. Thus, most completed annual assessments between October and January of each year. PROTECT pre-pandemic data was available from a data freeze in early October 2019.

### Data collected during COVID-19 (May 13–June 8, 2020)

The following information was collected during the pandemic.

#### Symptoms of COVID-19 infection

Participants were asked whether they had any of the main symptoms of COVID-19 in the last 2 weeks (which at the time were a new persistent cough for more than 24 h or a high temperature) or if they had been hospitalized with COVID-19 in the last 4 weeks.

#### Physical activity changes

Participants were asked about changes in their physical activity since March 2020. The data were categorized to identify people who reported a decrease in their level of physical activity and those who did not.

#### Physical illnesses

Participants were asked if they had any of the following conditions associated with moderately increased risk of severe illness from coronavirus: long-term respiratory illness, chronic heart disease, chronic kidney disease, liver disease, neurological disease, diabetes, illness affecting the spleen, weakened immune system, or BMI ≥ 40. They were also asked if they had any of the following conditions, which would require them to shield (high risk of severe illness from coronavirus): received an organ transplant and remain on ongoing immunosuppression medication, undergoing active chemotherapy or radiotherapy, cancer of the blood or bone marrow who are at any stage of treatment, severe chest conditions such as cystic fibrosis or severe asthma (requiring hospital admissions or course of steroid tablets), severe diseases of body systems. People were also asked if they had received a letter advising them to shield and if they answered yes, they were included in the high-risk group. These physical conditions were coded 0 (no relevant conditions); 1 (moderate-risk conditions); and 2 (high-risk conditions).

#### Loneliness

Loneliness was assessed using the 3-item UCLA loneliness scale (Hughes *et al.*, 2004). The questions ask how often the participant has felt a lack of companionship, left out, and isolated from others with the possible answers being “hardly ever”, “some of the time”, and “often”. Loneliness was treated as binary for this analysis, dichotomized into those experiencing any loneliness (i.e. rating at least “some of the time” on any question) and those experiencing none.

#### Finances

Participants were asked to respond yes or no to the question “Has the COVID-19 (coronavirus) pandemic had a negative impact on your finances?”

### Statistical methods

The statistical analyses were carried out in two stages.

In the full cohort, we first undertook a case-level analysis of PHQ-9 and GAD-7 rated in 2020 during the pandemic, categorizing both into a three-level factor (see above for cutoffs) representing no, mild, and moderate-to-severe symptoms. Differences in the proportions of current depression and anxiety levels by risk factor were analyzed using the χ^2^ test. We then undertook a descriptive analysis of the change in case-level proportions between 2019 and 2020.

For the second and principal analysis, we examined trajectories of PHQ-9 and GAD-7 between October 2015 (the start of the PROTECT study) and June 8, 2020. Initial analysis of PHQ-9 and GAD-7 total scores using linear mixed-effects models showed evidence of departure from the assumption of normally distributed residuals (see supplement). This could not be rectified by transformations and instead, we considered models for counts of symptoms. A zero-inflated negative binomial regression (ZINB) was chosen for each scale due to over-dispersion and evidence of excess zeros. ZINB models use a mixture model approach in which the population is assumed to consist of an at-risk subgroup, and a subgroup not at risk for PHQ-9 and GAD-7 symptoms during the study period (the source of the excess zeros). The model is comprised of two components: the first accounts for the distribution of symptoms in the at-risk population (negative binomial component) and the second is a logit model accounting for factors associated with membership of the non-risk subpopulation (zero-inflated component). A random intercept term was included to allow for correlations between repeated measurements on the same individual.

First, separate ZINB models were run for both PHQ-9 and GAD-7 for each individual risk factor (i.e. loneliness and physical activity, as well as the following socioeconomic variables: age group [under 70 and 70 and over], gender, psychiatric diagnosis history, education level, employment status, marital status, negative financial impact of the pandemic, and risk medical condition). Education, employment status, and marital status were all dummy coded. Linear and quadratic terms for time since study start and a 2020 indicator variable were added to estimate the effect of the pandemic on PHQ-9 and GAD-7 scores after removing any background trend in previous years. The zero-inflated components of the models did not include an interaction term between each risk factor and the year 2020 because models were not significantly improved by including one. Therefore, the zero-inflated component did not tell us anything specific about the effect of 2020 so for simplicity they are not reported here. Incidence rate ratios (IRR) were calculated to illustrate the incremental effect on PHQ-9 and GAD-7 scores of each risk factor in 2020 relative to those without the risk factor.

All statistically significant variables were included in the final adjusted model to assess which risk factors were independently associated with PHQ-9 and GAD-7. Predicted values from the adjusted final model were obtained and plotted for year 0 (study start, October 2015), 1 year, 2 years, and 3 years after study start, and during the pandemic (i.e. ~4.5 years after study start).

**Figure 1.**
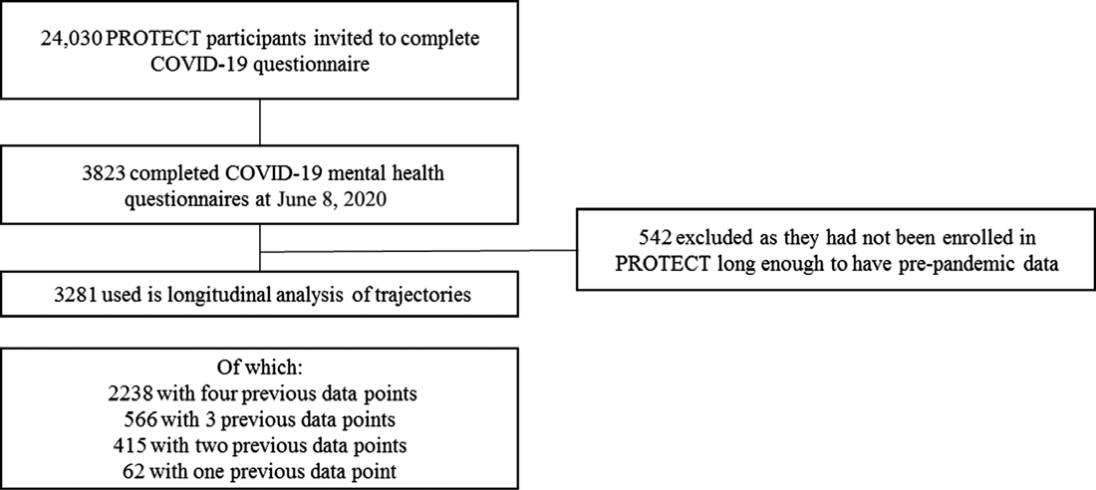
Consort chart.

Of the 3281 people who completed the COVID-19 mental health questionnaire in 2020, 2238 had 4 previous data points; 566 had 3; 415 had 2; and 62 had 1 (Figure 1). The distribution of assessment by month in each year is shown in the supplement.

Statistical analysis was undertaken in the R software environment for statistical computing. Longitudinal zero-inflated negative binomial regression models were fitted using the package glmmTMB (https://github.com/glmmTMB/glmmTMB).

### Role of the funding source

The funder had no role in any part of the project. The corresponding author had full access to all the data in the study and had final responsibility for the decision to submit for publication.

## Results

### Participants

In total, 3281 people completed the COVID-19 mental health questionnaire, 542 of these either joined PROTECT in May/June 2020 specifically to do the COVID-19 element or they joined PROTECT after the October 2019 data freeze so there was no pre-pandemic data available for this analysis. These were excluded, but there were no major differences in characteristics between the 3281 used in this analysis and the 542 excluded (see supplement). The characteristics of the sample analyzed are described in Table 1. The mean age in 2020 was 67 (standard deviation 6.5, range 55–96), around one-third had an undergraduate level education, 80% were female and 98% were White (because of the very low numbers of other ethnicities, ethnicity was not considered further in the analysis). These figures are similar to the wider 25,000 PROTECT study sample (Creese *et al.*, 2019). Twenty-six (.7%) people reported having a new continuous cough or high temperature in the last 2 weeks, a similar proportion (1%) reported a family member with these symptoms. One person in the sample had been hospitalized with COVID-19 in the last 4 weeks.

**Table 1.** Demographics characteristics for the whole sample

	total	%
**Age group**		
70 and over	1001	30
Under 70	2280	70
**Gender**		
Female	2610	80
Male	671	20
**Marital status**		
Married/civil partnership/cohabiting	2421	74
Widow/separated/divorce	615	19
Single	245	7
**Education**		
School–16	400	12
16–18	1006	31
Undergrad	1142	35
Postgrad	733	22
**Employment**		
Employed (full-time)	509	16
Employed (part-time)	569	17
Self-employed	280	9
Retired	1847	56
Unemployed	76	2
**Lifetime history of any psychiatric illness**		
No	2134	65
Yes	1147	35

## Risk factors and trajectories

### Case-level analysis

In the cross-sectional pandemic data, every variable except education level was associated with higher proportions of mild and moderate-to-severe depressive and anxiety symptoms (see supplement for proportions). Mild and moderate-to-severe anxiety cases were generally less common. All variables except education level and marital status were associated with higher proportions of anxiety cases.

We then compared case-level differences in 2019 with 2020. Overall, case-level estimates for moderate-to-severe symptoms were comparable across the 2 years. One-hundred and eighty-five (5.6%, 95% CI: 4.9–6.4) and 89 (2.7% 95% CI: 2.2–3.3) of 3281 people in 2020 had a PHQ-9 score of ≥10 and a GAD-7 score of ≥10, respectively. This is compared with 124 (4.1%, 95% CI: 3.5–5) and 66 (2.2%, 95% CI: 1.8–2.8), respectively, with moderate-to-severe symptoms in 2019 (*n* = 2959). There was a more pronounced difference in mild symptoms. In 2020, 634 (19%, 95% CI: 18–20.7) had mild depressive symptoms compared with 392 (13.2%, 95% CI: 12.1–14.5) in 2019. Similarly, 415 people had mild anxiety symptoms in 2020 (12.6%, 95% CI: 11.6–13.8) compared with 276 in 2019 (9.3%, 95% CI: 8.3–10.4).

### Trajectories of PHQ-9 and GAD-7 scores

The results from the ZINB models for each individual risk factor are shown in supplementary data. Loneliness, decreased physical activity, being a woman, and being retired were all associated with significant worsening of depressive symptoms in 2020. Similarly, loneliness, decreased physical activity, and being a woman were also associated with worsening GAD-7 scores in 2020. Not being in full-time employment was associated with a greater worsening of GAD-7 score relative to being full-time employed. Both those with a psychiatric history and those without experienced worsening symptoms during the pandemic, but the change was relatively higher in the no history group. The absolute GAD-7 score for people with a psychiatric diagnosis was consistently higher throughout the entire study period.

For the final adjusted model of PHQ-9 trajectory, loneliness, activity level, gender, and retirement status were all included as covariates. For the GAD-7 adjusted model, loneliness, physical activity, gender, full-time employment status, and history of the psychiatric condition were included as covariates.

**Figure 2.**
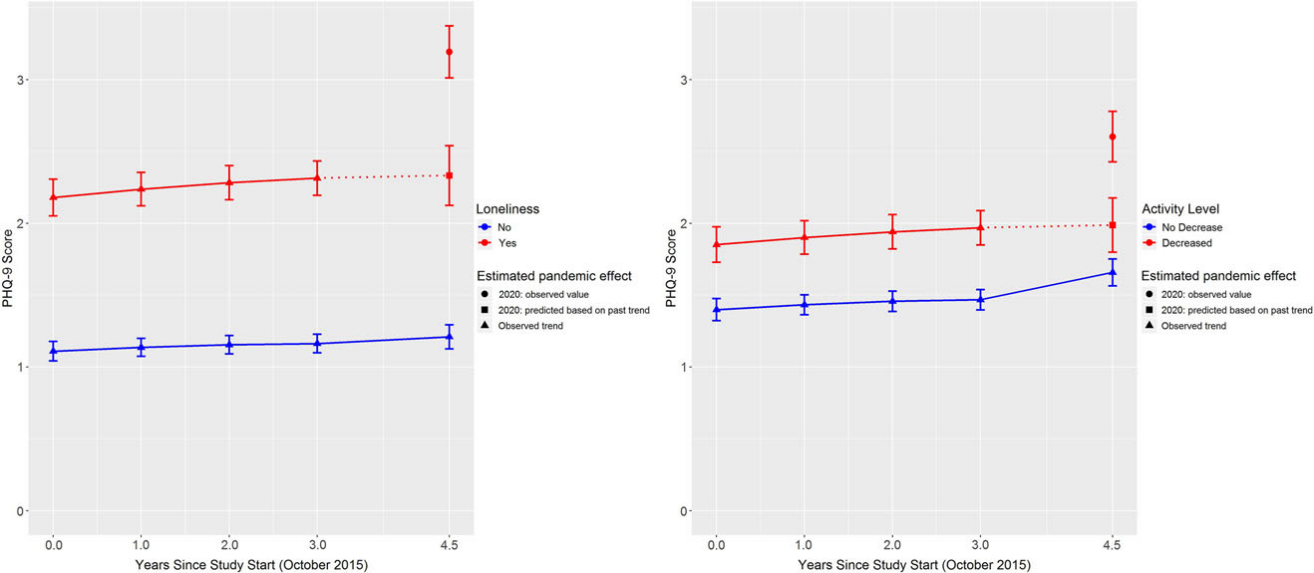
Trajectories of predicted PHQ-9 scores from zero-inflated negative binomial regression models for loneliness and physical activity. Error bars are 95% confidence intervals.

**Figure 3.**
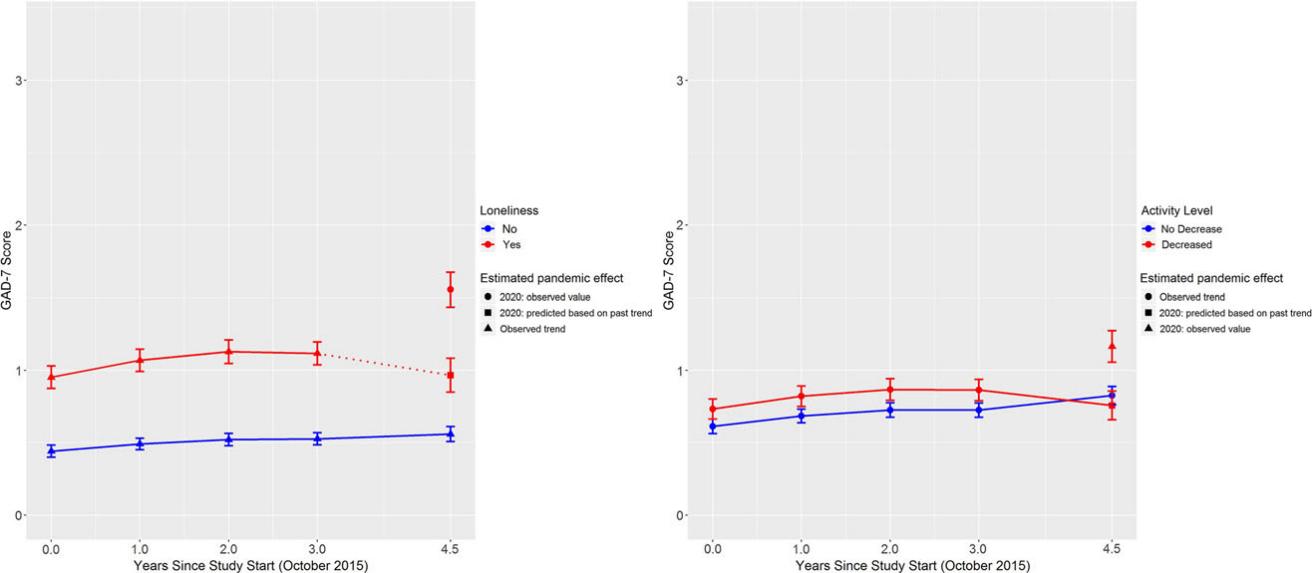
Trajectories of predicted GAD-7 scores from zero-inflated negative binomial regression models for loneliness and physical activity. Error bars are 95% confidence intervals.

Results from the adjusted models are shown in Table 2 and predicted adjusted PHQ-9 and GAD-7 scores for each time point are provided in full in the supplement along with their 95% confidence intervals, plots of these predicted values for loneliness and physical activity are shown in Figures 2 and 3.

**Table 2.** Adjusted negative binomial regression component of ZINB models of PHQ-9 and GAD-7. Regression coefficients represent the effect of the 2020 indicator variable on scores (rows in bold) and the interaction between each risk factor and the 2020 indicator (all other rows)

	**PHQ-9**			
risk factor	irr	l 95% ci	u 95% ci	p
**Year 2020**	.**97**	.**88**	**1.08**	.**64**
Loneliness*Year 2020	1.29	1.21	1.38	<.0001
Activity level decreased*Year 2020	1.15	1.08	1.22	<.0001
Women*Year 2020	1.13	1.04	1.23	.004
Retired*Year 2020	1.11	1.04	1.17	.001
	**GAD-7**			
**Year 2020**	**1.24**	**1.08**	**1.43**	.**003**
Loneliness*Year 2020	1.37	1.25	1.50	<.0001
Activity level decreased*Year 2020	1.20	1.10	1.30	<.0001
Women*Year 2020	1.22	1.09	1.36	.0004
Full-time employed*Year 2020	.88	.78	.98	.02
History of psychiatric condition*Year 2020	.85	.78	.92	<.0001

Abbreviations: ZINB, zero-inflated negative binomial regression; IRR, incidence rate ratio.

#### Loneliness

In 2020, the difference in PHQ-9 scores between the lonely and the not lonely groups was 29% greater than in previous years (IRR = 1.29, 95% CI: 1.21–1.38, *p* < .0001). Prior to 2020, people rated as lonely scored approximately 1 point higher than those rated not lonely (Figure 2). In 2020 (4.5 years after study start), the difference between the two groups was ~2 points (Cohen’s *d* = .42), with PHQ-9 score increasing to 3.23 (95% CI: 3.01–3.44) among those who reported loneliness and remaining stable (1.22, 95% CI: 1.12–1.32) for those not reporting loneliness. In other words, about 50% of the difference in PHQ-9 score between loneliness and no loneliness during the pandemic was accounted for by the general higher burden of symptoms associated with being lonely. For context, this means that in 2020, people who were lonely reported either a new PHQ-9 symptom for several days of the last 2 weeks or a worsening of an existing symptom to more than half the days in the last 2 weeks.

For GAD-7, in 2020, symptoms were 37% worse in those who rated as lonely relative to the not lonely group, than in previous years (IRR = 1.37, 95% CI: 1.25–1.50, *p* < .0001). Among those with no loneliness, GAD-7 total score was .5 across all years (Figure 3). For those with loneliness, GAD-7 score was .5 higher (at around 1) in years prior to 2020 compared to the not lonely group, but in 2020, the score increased to 1.55 (95% CI: 1.43–1.67, Cohen’s *d* = .23). Again, the pandemic accounted for around 50% of the difference in GAD-7 scores attributable to loneliness in 2020.

#### Physical activity

In 2020, the differences in PHQ-9 and GAD-7 scores between those with decreased physical activity and those without were 15% and 20% higher than in previous years (IRR = 1.15, 95% CI: 1.08–1.22, *p* < .0001 and IRR = 1.20, 95% CI: 1.10–1.30, *p* < .0001, respectively). The general trajectory of PHQ-9 and the difference in scores between those reporting a decrease in physical activity, and those not, was similar to loneliness, although the absolute scores were smaller (Figure 2). That is, there was around a .5 point difference in the years prior to 2020 and 1 point difference in 2020 (decreased physical activity: 2.60, 95% CI: 2.43–2.78, no decrease: 1.66, 95% CI: 1.56–1.75, Cohen’s *d* = .37). Similar to loneliness again, GAD-7 score was modestly higher for people with decreased physical activity in the years prior to 2020 (Figure 3). However, in 2020, GAD-7 score increased to 1.17 (95% CI 1.06–1.27) among those reporting decreased physical activity, which compares with .83 for those with no decrease in physical activity (95% CI: .76–.89), a Cohen’s *d* of .14.

#### Gender, employment status, and psychiatric history

In 2020, the differences in PHQ-9 score between women and men and being retired and not retried were greater than in previous years (IRR = 1.13, 95% CI: 1.04–1.23, *p* = .004; IRR = 1.11, 95% CI: 1.04–1.17, *p* = .001). Similarly, the difference in GAD-7 scores between women and men was also greater in 2020 than in previous years (IRR = 1.22, 95% CI: 1.09–1.36, *p* = .0004). Having a history of a psychiatric condition was associated with a relatively lesser increase in GAD-7 score in 2020 compared to those without a history of psychiatric diagnosis (IRR = .85, 95% CI: .78−.92, *p* < .0001). The absolute GAD-7 score for people with a psychiatric history was higher in 2020 and all years prior than those without (1.49 [95% CI: 1.36–1.63] vs .72 [95% CI: .67–.78], see supplement). Similarly, full-time employment was associated with a more stable GAD-7 score, with a relative worsening in 2020 observed for those not in full-time employment, although the absolute values of PHQ-9 and GAD-7 were higher for people in full-time employment (IRR = .88, 95% CI: .78–.98, *p* = .02, see supplement) (Table 2).

## Discussion

To our knowledge, this is the first longitudinal study to focus specifically on the links between loneliness, physical activity, and mental health during the COVID-19 pandemic with longitudinal data also pertaining to pre-pandemic mental health. Overall, in a cohort aged between 55 and 96, there was an increase in the proportion of people with mild depressive symptoms from 13.2% in 2019 to 19% in 2020 and an increase in the proportion of people with mild anxiety symptoms (from 9.3% to 12.6%). The proportions of people with moderate-to-severe symptoms were comparable. Both loneliness and decreased physical activity were associated with worse mental health in 2020 compared to previous years. This suggests that the association observed in 2020 was not solely due to a longer standing relationship between current loneliness, physical activity, and mental health before 2020, overcoming an important limitation of previous cross-sectional studies with a measurement taken only during the pandemic (Frenkel-Yosef *et al.*, 2020). Our data also show that the impact of the pandemic on mental health would have been overestimated without the longitudinal perspective, bringing new insight to these established mental health risk factors and in line with other recent findings (Banks and Xu, 2020).

Around half of the sample reported some degree of loneliness during the pandemic. Loneliness was associated with a 1 point higher score on the PHQ-9 between 2015 and 2019 compared to people who did not report loneliness, but this difference doubled to 2 points during the pandemic. In contrast, there was no worsening of mental health symptoms for people who did not report loneliness. Over one-third of the sample reported decreased physical activity during the pandemic. The effect on PHQ-9 scores was more modest than that of loneliness, but was nevertheless associated with a worsening of symptoms. There were also statistically significant increases in GAD-7 scores for both loneliness and decreased physical activity though the absolute scores were smaller than for PHQ-9. For context, the increases in PHQ-9 can be interpreted as the emergence of a new symptom, or an existing symptom increasing in frequency to more than half the days in the last 2 weeks. This is a relatively modest increase, but an important observation given the established links between loneliness, physical activity, and mental health and given that it occurred within only the first 2 months of the UK lockdown. More longitudinal data through the later stages of the pandemic will help elucidate whether this upward trend is sustained or whether symptoms resolve. Collectively, these findings emphasize the potential impact of finding novel solutions to tackle loneliness and decreased physical activity during the pandemic and underscore the important general relationship between the two and mental health (Age UK, no date; Haskell *et al.*, 2009; Killgore *et al.*, 2020).

Of the socioeconomic and demographic variables analyzed, both being a woman, being retired, and not being full-time employed were associated with pandemic-specific worsening in mental health, in line with the previous UK representatively sampled studies (Fancourt *et al.*, 2020; Pierce *et al.*, 2020). While our data do not show any increase in mental health symptoms related to the pandemic having a negative financial impact, we believe it would be premature to rule out an effect of this variable on mental health; first because the economic impact of the pandemic has not yet fully taken hold and second because we note other large representative surveys have reported clear links (Frank *et al.*, 2020; Pierce *et al.*, 2020). Finally, similar to other studies, we found no evidence that having a medical condition, which is associated with increased risk of severe COVID-19 was associated with worsening symptoms of depression and anxiety (Pierce *et al.*, 2020).

### Limitations

One important limitation is the potential for bias in an on-line self-selecting sample. In particular, we note the overrepresentation of women, White British people, and those with a higher education, which means our findings may not be generalizable. However, because our analysis is focused on longitudinal patterns rather than prevalence, there is still merit in identifying these trends within this sample. The second limitation is determining causation, a pervasive issue in observational studies. Because the loneliness and physical activity questions were only asked during the pandemic, it may be the case that worse mental health drove a decrease in physical activity and an increase in loneliness. The wider literature has highlighted a causal relationship between higher physical activity levels and lower risk for major depressive disorder (but no causal relationship for the reverse) so in the context of this evidence, it would be reasonable to hypothesize that maintaining physical activity during the pandemic may mitigate the risk of mental health deterioration (Choi *et al.*, 2019). A large randomized control trial would be needed to assess this but our findings pave the way for robust intervention testing. We are not aware of any studies, which have conclusively shown a causal directional link between loneliness and mental health, but the well-established link between the two is one of the reasons why loneliness is a critical policy area in the UK and internationally. Here, we are able to show that for the first time that the association between loneliness and worse mental health is not solely due to those who are currently lonely having long-standing worse mental health; there is a specific effect of 2020 in this sample, which is an important advance over previous cross-sectional studies. The effect of the pandemic was modeled by an interaction term between loneliness and the year 2020 and we modeled the 2020 trend without the interaction term to show what a continuation of the trend of past years may look like. We have, therefore, concluded that the increase we observed in 2020 is attributable to the pandemic. We would argue this is a reasonable conclusion given that our 4 years of measurements prior to the pandemic show symptoms to be generally flat. However, we cannot rule out that there may also be other factors influencing mental health changes which we could not measure, longer term data through the later phases of the pandemic will help answer this question.

In conclusion, in this large longitudinally studied sample exploring mental health effects of the COVID-19 pandemic in middle-aged and older people in the UK, we found that loneliness and decreased physical activity were both associated with worse mental health and that this was distinct from the general relationship between these two risk factors and poor mental health. Our study provides robust evidence in support of targeted interventions – which may include resilience training, physical activity, or strategies to reduce loneliness – to improve the mental health of people in mid to late life in the subsequent waves of the pandemic.

## Conflict of interests

The authors declare no competing interests. The views expressed are those of the author(s) and not necessarily those of the NHS, the NIHR, or the Department of Health and Social Care or Public Health England.

## Supplementary material

For supplementary material accompanying this paper visit http://dx.doi.org/10.1017/S1041610220004135. click here to view supplementary material
