# Supplementary material for: Loneliness, physical activity, and mental health during COVID-19: a longitudinal analysis of depression and anxiety in adults over the age of 50 between 2015 and 2020
Source: Int Psychogeriatr. 2020 Dec 17:1–10. doi: 10.1017/S1041610220004135 (PMC7985900; doi:10.1017/S1041610220004135)
Supplement: Supplementary file 1 [file S1041610220004135sup001.docx]

**Figure 1 Histogram of PHQ-9 score during pandemic**

**Figure 2 Histogram of GAD-7 score during pandemic**

**Table 1: Characteristic of sample analysed (pre-pandemic data) and sample with no pre-pandemic data (therefore excluded)**

|  | **No pre-pandemic data** | | **Pre-pandemic data** | |
| --- | --- | --- | --- | --- |
|  | **N** | **%** | **N** | **%** |
| **Age** |  |  |  |  |
| 70 or over | 154 | 28 | 1001 | 31 |
| 55-69 | 388 | 72 | 2280 | 69 |
| **Gender** |  |  |  |  |
| Female | 434 | 80 | 2610 | 80 |
| Male | 108 | 20 | 671 | 20 |
| **Education Level** |  |  |  |  |
| School to 16 | 64 | 12 | 400 | 12 |
| 16 to 18 | 149 | 27 | 1006 | 31 |
| Undergraduate | 208 | 38 | 1142 | 35 |
| Post graduate | 121 | 22 | 733 | 22 |
| **Marital status** |  |  |  |  |
| Married/ Civil Partnership/Co-habiting | 392 | 72 | 2421 | 74 |
| Widow/Separated /Divorce | 111 | 20 | 615 | 19 |
| Single | 39 | 7 | 245 | 7 |
| **Employment status** |  |  |  |  |
| Employed (full-time) | 100 | 18 | 509 | 16 |
| Employed (part-time) | 109 | 20 | 569 | 17 |
| Self-employed | 55 | 10 | 280 | 9 |
| Retired | 259 | 48 | 1847 | 56 |
| Unemployed | 19 | 4 | 76 | 2 |
| **PHQ-9** |  |  |  |  |
| None | 375 | 69 | 2462 | 75 |
| Mild | 123 | 23 | 634 | 19 |
| Moderate-to-severe | 44 | 8 | 185 | 6 |
| **GAD-7** |  |  |  |  |
| None | 446 | 82 | 2777 | 85 |
| Mild | 75 | 14 | 415 | 13 |
| Moderate-to-severe | 21 | 4 | 89 | 3 |

**Table 2: distribution of assessments by month and year**

|  | **Month** | | | | | | | | | | | |
| --- | --- | --- | --- | --- | --- | --- | --- | --- | --- | --- | --- | --- |
|  | **Jan** | **Feb** | **Mar** | **Apr** | **May** | **Jun** | **Jul** | **Aug** | **Sep** | **Oct** | **Nov** | **Dec** |
| **2015** | - | - | - | - | - | - | - | - | - | 145 | 1679 | 106 |
| **2016** | 62 | 47 | 47 | 45 | 179 | 75 | 55 | 58 | 32 | 3 | 376 | 1360 |
| **2017** | 180 | 96 | 106 | 80 | 194 | 112 | 106 | 91 | 122 | 111 | 1297 | 253 |
| **2018** | 178 | 119 | 135 | 100 | 269 | 154 | 157 | 118 | 129 | 121 | 78 | 8 |
| **2019** | 1437 | 367 | 175 | 100 | 281 | 158 | 171 | 108 | 144 | 18 | - | - |
| **2020** | - | - | - | - | 2616 | 665 | - | - | - | - | - | - |

**Table 3 Risk factors by depression and anxiety case status in the whole sample during pandemic (N=3,281)**

|  | **Depressive symptoms during pandemic (n, %)** | | | | | | | |  | **Anxiety symptoms during pandemic (n, %)** | | | | | | | |
| --- | --- | --- | --- | --- | --- | --- | --- | --- | --- | --- | --- | --- | --- | --- | --- | --- | --- |
|  | **None** | | **Mild** | | **Moderate to Severe** | | **Total** | p |  | **None** | | **Mild** | | **Moderate to Severe** | | **Total** | p |
| Loneliness No | 1476 | 88 | 174 | 10 | 20 | 1 | 1670 | <0.001 |  | 1554 | 93 | 107 | 6 | 9 | 1 | 1670 | <0.001 |
| Loneliness Yes | 986 | 61 | 460 | 29 | 165 | 10 | 1611 |  |  | 1223 | 76 | 308 | 19 | 80 | 5 | 1611 |  |
|  |  |  |  |  |  |  |  |  |  |  |  |  |  |  |  |  |  |
| Activity Levels Same | 1705 | 81 | 344 | 16 | 68 | 3 | 2117 | <0.001 |  | 1855 | 88 | 225 | 11 | 37 | 2 | 2117 | <0.001 |
| Activity Levels Decreased | 757 | 65 | 290 | 25 | 117 | 10 | 1164 |  |  | 922 | 79 | 190 | 16 | 52 | 4 | 1164 |  |
|  |  |  |  |  |  |  |  |  |  |  |  |  |  |  |  |  |  |
| No Negative Financial Impact | 1972 | 77 | 470 | 18 | 122 | 5 | 2564 | <0.001 |  | 2209 | 86 | 295 | 12 | 60 | 2 | 2564 | <0.001 |
| Negative Financial Impact | 490 | 68 | 164 | 23 | 63 | 9 | 717 |  |  | 568 | 79 | 120 | 17 | 29 | 4 | 717 |  |
|  |  |  |  |  |  |  |  |  |  |  |  |  |  |  |  |  |  |
| No Risk Conditions | 1960 | 76 | 489 | 19 | 118 | 5 | 2567 | <0.001 |  | 2187 | 85 | 327 | 13 | 53 | 2 | 2567 | <0.001 |
| Moderate Risk Conditions | 447 | 70 | 130 | 20 | 62 | 10 | 639 |  |  | 527 | 82 | 79 | 12 | 33 | 5 | 639 |  |
| High Risk Conditions | 55 | 73 | 15 | 20 | 5 | 7 | 75 |  |  | 63 | 84 | 9 | 12 | 3 | 4 | 75 |  |
|  |  |  |  |  |  |  |  |  |  |  |  |  |  |  |  |  |  |
| Female | 1908 | 73 | 544 | 21 | 158 | 6 | 2610 | <0.001 |  | 871 | 87 | 108 | 11 | 22 | 2 | 1001 | 0.04 |
| Male | 554 | 83 | 90 | 13 | 27 | 4 | 671 |  |  | 1906 | 84 | 307 | 13 | 67 | 3 | 2280 |  |
|  |  |  |  |  |  |  |  |  |  |  |  |  |  |  |  |  |  |
| 70 and over | 788 | 79 | 169 | 17 | 44 | 4 | 1001 | 0.004 |  | 2161 | 83 | 367 | 14 | 82 | 3 | 2610 | <0.001 |
| 55-69 | 1674 | 73 | 465 | 20 | 141 | 6 | 2280 |  |  | 616 | 92 | 48 | 7 | 7 | 1 | 671 |  |
|  |  |  |  |  |  |  |  |  |  |  |  |  |  |  |  |  |  |
| Married/ Civil Partnership/Co-habiting | 1863 | 77 | 439 | 18 | 119 | 5 | 2421 | <0.001 |  | 2053 | 85 | 300 | 12 | 68 | 3 | 2421 | 0.48 |
| Widow/Separated /Divorce | 432 | 70 | 131 | 21 | 52 | 8 | 615 |  |  | 510 | 83 | 89 | 14 | 16 | 3 | 615 |  |
| Single | 167 | 68 | 64 | 26 | 14 | 6 | 245 |  |  | 214 | 87 | 26 | 11 | 5 | 2 | 245 |  |
|  |  |  |  |  |  |  |  |  |  |  |  |  |  |  |  |  |  |
| School to 16 | 299 | 75 | 72 | 18 | 29 | 7 | 400 | 0.17 |  | 341 | 85 | 49 | 12 | 10 | 3 | 400 | 0.59 |
| 16 to 18 | 738 | 73 | 201 | 20 | 67 | 7 | 1006 |  |  | 847 | 84 | 133 | 13 | 26 | 3 | 1006 |  |
| Undergrad | 864 | 76 | 221 | 19 | 57 | 5 | 1142 |  |  | 971 | 85 | 139 | 12 | 32 | 3 | 1142 |  |
| Post-grad | 449 | 75 | 121 | 20 | 28 | 5 | 598 |  |  | 496 | 83 | 85 | 14 | 17 | 3 | 598 |  |
| Doctorate | 112 | 83 | 19 | 14 | 4 | 3 | 135 |  |  | 122 | 90 | 9 | 7 | 4 | 3 | 135 |  |
|  |  |  |  |  |  |  |  |  |  |  |  |  |  |  |  |  |  |
| Employed (full-time) | 380 | 75 | 100 | 20 | 29 | 6 | 509 | <0.001 |  | 428 | 84 | 68 | 13 | 13 | 3 | 509 | <0.001 |
| Employed (part-time) | 411 | 72 | 124 | 22 | 34 | 6 | 569 |  |  | 463 | 81 | 88 | 15 | 18 | 3 | 569 |  |
| Self-employed | 215 | 77 | 51 | 18 | 14 | 5 | 280 |  |  | 235 | 84 | 42 | 15 | 3 | 1 | 280 |  |
| Retired | 1419 | 77 | 331 | 18 | 97 | 5 | 1847 |  |  | 1597 | 86 | 201 | 11 | 49 | 3 | 1847 |  |
| Unemployed | 37 | 49 | 28 | 37 | 11 | 14 | 76 |  |  | 54 | 71 | 16 | 21 | 6 | 8 | 76 |  |
|  |  |  |  |  |  |  |  |  |  |  |  |  |  |  |  |  |  |
| No history of psychiatric condition | 1774 | 83 | 307 | 14 | 53 | 2 | 2134 | <0.001 |  | 1933 | 91 | 184 | 9 | 17 | 1 | 2137 | <0.001 |
| History of psychiatric condition | 688 | 60 | 327 | 29 | 132 | 12 | 1147 |  |  | 844 | 74 | 231 | 20 | 72 | 6 | 1147 |  |

**Table 4 Negative binomial regression component of ZINB models of PHQ-9 and GAD-7 trajectories for individual risk factors. Values are not adjusted for other variables. Regression coefficients represent the interaction between each risk factor and the 2020 indicator**

|  | **PHQ-9** | | | |  | **GAD-7** | | | |
| --- | --- | --- | --- | --- | --- | --- | --- | --- | --- |
|  | **IRR** | **L 95% CI** | **U 95% CI** | **P** |  | **IRR** | **L 95% CI** | **U 95% CI** | **P** |
| **Loneliness** |  |  |  |  |  |  |  |  |  |
| Loneliness*Year 2020 | 1.32 | 1.23 | 1.41 | <0.001 |  | 1.39 | 1.27 | 1.52 | <0.001 |
| **Activity Level Change** |  |  |  |  |  |  |  |  |  |
| Decreased activity*Year 2020 | 1.18 | 1.11 | 1.25 | <0.001 |  | 1.23 | 1.13 | 1.34 | <0.001 |
| **Negative financial impact** |  |  |  |  |  |  |  |  |  |
| Negative financial impact*Year 2020 | 1.07 | 1.00 | 1.15 | 0.05 |  | 1.08 | 0.98 | 1.18 | 0.11 |
| **Risk medical conditions** |  |  |  |  |  |  |  |  |  |
| Moderate risk conditions*Year 2020 | 0.93 | 0.87 | 1.00 | 0.05 |  | 1.00 | 0.90 | 1.10 | 0.98 |
| High risk conditions*Year 2020 | 1.09 | 0.91 | 1.31 | 0.34 |  | 1.20 | 0.92 | 1.56 | 0.19 |
| **Age Group** |  |  |  |  |  |  |  |  |  |
| 55-69*Year 2020 | 0.95 | 0.89 | 1.02 | 0.13 |  | 0.97 | 0.89 | 1.06 | 0.53 |
| **Gender** |  |  |  |  |  |  |  |  |  |
| Women*Year 2020 | 1.14 | 1.05 | 1.25 | 0.002 |  | 1.22 | 1.09 | 1.36 | 0.00 |
| **Psychiatric history** |  |  |  |  |  |  |  |  |  |
| History of psychiatric condition*Year 2020 | 0.96 | 0.90 | 1.02 | 0.177 |  | 0.91 | 0.84 | 0.99 | 0.03 |
| **Education** |  |  |  |  |  |  |  |  |  |
| School to 16*Year 2020 | 0.98 | 0.89 | 1.07 | 0.61 |  | 0.93 | 0.82 | 1.05 | 0.26 |
| 16 to 18*Year 2020 | 0.98 | 0.90 | 1.07 | 0.66 |  | 1.00 | 0.88 | 1.13 | 0.94 |
| Undergraduate*Year 2020 | 0.99 | 0.93 | 1.05 | 0.68 |  | 1.01 | 0.92 | 1.12 | 0.78 |
| Post-graduate*Year 2020 | 1.05 | 0.97 | 1.13 | 0.20 |  | 1.06 | 0.97 | 1.15 | 0.22 |
| **Employment** |  |  |  |  |  |  |  |  |  |
| Employed (full-time) *Year 2020 | 0.93 | 0.86 | 1.01 | 0.10 |  | 0.87 | 0.78 | 0.98 | 0.02 |
| Employed (part-time) *Year 2020 | 0.98 | 0.91 | 1.06 | 0.68 |  | 1.04 | 0.94 | 1.15 | 0.47 |
| Self-employed*Year 2020 | 0.91 | 0.82 | 1.02 | 0.10 |  | 0.94 | 0.81 | 1.09 | 0.42 |
| Retired*Year 2020 | 1.10 | 1.04 | 1.17 | 0.002 |  | 1.08 | 0.99 | 1.17 | 0.08 |
| Unemployed*Year 2020 | 0.86 | 0.73 | 1.01 | 0.07 |  | 0.98 | 0.80 | 1.19 | 0.81 |
| **Marital status** |  |  |  |  |  |  |  |  |  |
| Married/Civil Partnership/Co-habiting*Year 2020 | 1.01 | 0.94 | 1.07 | 0.85 |  | 1.04 | 0.95 | 1.14 | 0.36 |
| Widow/Separated /Divorce*Year 2020 | 1.08 | 0.96 | 1.21 | 0.20 |  | 1.13 | 0.95 | 1.34 | 0.16 |
| Single*Year 2020 | 1.03 | 0.82 | 1.30 | 0.77 |  | 0.94 | 0.69 | 1.29 | 0.70 |

ZINB: zero-inflated negative binomial regression; IRR: incidence rate ratio

**Table 5: Predicted adjusted PHQ-9 scores by year and by risk factor**

| Year | Yr2020 | PHQ-9 | UCI | LCI | RiskFactorName | Group |
| --- | --- | --- | --- | --- | --- | --- |
| 0 | 0 | 1.15 | 1.22 | 1.07 | No | Loneliness |
| 1 | 0 | 1.17 | 1.25 | 1.1 | No | Loneliness |
| 2 | 0 | 1.19 | 1.27 | 1.12 | No | Loneliness |
| 3 | 0 | 1.2 | 1.28 | 1.12 | No | Loneliness |
| 4.5 | 1 | 1.22 | 1.32 | 1.12 | No | Loneliness |
| 0 | 0 | 2.25 | 2.4 | 2.1 | Yes | Loneliness |
| 1 | 0 | 2.31 | 2.45 | 2.17 | Yes | Loneliness |
| 2 | 0 | 2.36 | 2.5 | 2.22 | Yes | Loneliness |
| 3 | 0 | 2.39 | 2.54 | 2.25 | Yes | Loneliness |
| 4.5 | 0 | 2.41 | 2.64 | 2.18 | Yes | Loneliness |
| 4.5 | 1 | 3.23 | 3.44 | 3.01 | Yes | Loneliness |
| 0 | 0 | 1.4 | 1.48 | 1.32 | Not decreased | Activity Decreased |
| 1 | 0 | 1.43 | 1.5 | 1.36 | Not decreased | Activity Decreased |
| 2 | 0 | 1.46 | 1.53 | 1.39 | Not decreased | Activity Decreased |
| 3 | 0 | 1.47 | 1.54 | 1.4 | Not decreased | Activity Decreased |
| 4.5 | 1 | 1.66 | 1.75 | 1.56 | Not decreased | Activity Decreased |
| 0 | 0 | 1.85 | 1.98 | 1.73 | Decreased | Activity Decreased |
| 1 | 0 | 1.9 | 2.02 | 1.79 | Decreased | Activity Decreased |
| 2 | 0 | 1.94 | 2.06 | 1.82 | Decreased | Activity Decreased |
| 3 | 0 | 1.97 | 2.09 | 1.85 | Decreased | Activity Decreased |
| 4.5 | 0 | 1.99 | 2.18 | 1.8 | Decreased | Activity Decreased |
| 4.5 | 1 | 2.6 | 2.78 | 2.43 | Decreased | Activity Decreased |
| 0 | 0 | 1.76 | 1.87 | 1.65 | No | Retired |
| 1 | 0 | 1.8 | 1.9 | 1.7 | No | Retired |
| 2 | 0 | 1.83 | 1.94 | 1.73 | No | Retired |
| 3 | 0 | 1.85 | 1.96 | 1.75 | No | Retired |
| 4.5 | 1 | 2.1 | 2.23 | 1.96 | No | Retired |
| 0 | 0 | 1.4 | 1.48 | 1.32 | Yes | Retired |
| 1 | 0 | 1.44 | 1.51 | 1.36 | Yes | Retired |
| 2 | 0 | 1.47 | 1.54 | 1.39 | Yes | Retired |
| 3 | 0 | 1.48 | 1.56 | 1.41 | Yes | Retired |
| 4.5 | 0 | 1.49 | 1.62 | 1.36 | Yes | Retired |
| 4.5 | 1 | 1.87 | 1.97 | 1.76 | Yes | Retired |
| 0 | 0 | 1.57 | 1.65 | 1.49 | Women | Gender |
| 1 | 0 | 1.61 | 1.68 | 1.54 | Women | Gender |
| 2 | 0 | 1.64 | 1.71 | 1.57 | Women | Gender |
| 3 | 0 | 1.66 | 1.74 | 1.59 | Women | Gender |
| 4.5 | 1 | 2.05 | 2.15 | 1.95 | Women | Gender |
| 0 | 0 | 1.46 | 1.58 | 1.33 | Men | Gender |
| 1 | 0 | 1.49 | 1.62 | 1.37 | Men | Gender |
| 2 | 0 | 1.51 | 1.64 | 1.39 | Men | Gender |
| 3 | 0 | 1.52 | 1.65 | 1.39 | Men | Gender |
| 4.5 | 0 | 1.49 | 1.66 | 1.32 | Men | Gender |
| 4.5 | 1 | 1.62 | 1.79 | 1.46 | Men | Gender |

**Table 6: Predicted adjusted GAD-7 scores by year and by risk factor**

| Year | Yr2020 | GAD-7 | UCI | LCI | RiskFactorName | Group |
| --- | --- | --- | --- | --- | --- | --- |
| 0 | 0 | 0.44 | 0.48 | 0.4 | No | Loneliness |
| 1 | 0 | 0.49 | 0.53 | 0.45 | No | Loneliness |
| 2 | 0 | 0.52 | 0.56 | 0.48 | No | Loneliness |
| 3 | 0 | 0.53 | 0.57 | 0.48 | No | Loneliness |
| 4.5 | 1 | 0.56 | 0.61 | 0.51 | No | Loneliness |
| 0 | 0 | 0.95 | 1.03 | 0.87 | Yes | Loneliness |
| 1 | 0 | 1.07 | 1.15 | 0.99 | Yes | Loneliness |
| 2 | 0 | 1.13 | 1.21 | 1.05 | Yes | Loneliness |
| 3 | 0 | 1.12 | 1.2 | 1.04 | Yes | Loneliness |
| 4.5 | 0 | 0.97 | 1.08 | 0.85 | Yes | Loneliness |
| 4.5 | 1 | 1.55 | 1.67 | 1.43 | Yes | Loneliness |
| 0 | 0 | 0.61 | 0.66 | 0.56 | Not decreased | Activity Decreased |
| 1 | 0 | 0.69 | 0.73 | 0.64 | Not decreased | Activity Decreased |
| 2 | 0 | 0.73 | 0.78 | 0.67 | Not decreased | Activity Decreased |
| 3 | 0 | 0.73 | 0.78 | 0.68 | Not decreased | Activity Decreased |
| 4.5 | 1 | 0.83 | 0.89 | 0.76 | Not decreased | Activity Decreased |
| 0 | 0 | 0.73 | 0.8 | 0.66 | Decreased | Activity Decreased |
| 1 | 0 | 0.82 | 0.89 | 0.75 | Decreased | Activity Decreased |
| 2 | 0 | 0.87 | 0.94 | 0.79 | Decreased | Activity Decreased |
| 3 | 0 | 0.86 | 0.94 | 0.79 | Decreased | Activity Decreased |
| 4.5 | 0 | 0.76 | 0.86 | 0.66 | Decreased | Activity Decreased |
| 4.5 | 1 | 1.17 | 1.27 | 1.06 | Decreased | Activity Decreased |
| 0 | 0 | 0.63 | 0.68 | 0.59 | No | Full Time |
| 1 | 0 | 0.71 | 0.75 | 0.66 | No | Full Time |
| 2 | 0 | 0.75 | 0.79 | 0.7 | No | Full Time |
| 3 | 0 | 0.75 | 0.79 | 0.7 | No | Full Time |
| 4.5 | 1 | 0.92 | 0.98 | 0.86 | No | Full Time |
| 0 | 0 | 0.78 | 0.89 | 0.68 | Yes | Full Time |
| 1 | 0 | 0.88 | 0.99 | 0.77 | Yes | Full Time |
| 2 | 0 | 0.93 | 1.04 | 0.81 | Yes | Full Time |
| 3 | 0 | 0.92 | 1.04 | 0.81 | Yes | Full Time |
| 4.5 | 0 | 0.82 | 0.95 | 0.69 | Yes | Full Time |
| 4.5 | 1 | 1 | 1.14 | 0.86 | Yes | Full Time |
| 0 | 0 | 0.69 | 0.74 | 0.63 | Women | Gender |
| 1 | 0 | 0.77 | 0.81 | 0.72 | Women | Gender |
| 2 | 0 | 0.81 | 0.86 | 0.76 | Women | Gender |
| 3 | 0 | 0.81 | 0.86 | 0.76 | Women | Gender |
| 4.5 | 1 | 1.03 | 1.1 | 0.96 | Women | Gender |
| 0 | 0 | 0.54 | 0.6 | 0.47 | Men | Gender |
| 1 | 0 | 0.6 | 0.67 | 0.53 | Men | Gender |
| 2 | 0 | 0.64 | 0.71 | 0.56 | Men | Gender |
| 3 | 0 | 0.63 | 0.71 | 0.56 | Men | Gender |
| 4.5 | 0 | 0.55 | 0.63 | 0.47 | Men | Gender |
| 4.5 | 1 | 0.64 | 0.73 | 0.56 | Men | Gender |
| 0 | 0 | 0.48 | 0.52 | 0.44 | No | Psychiatric Diagnosis |
| 1 | 0 | 0.53 | 0.57 | 0.49 | No | Psychiatric Diagnosis |
| 2 | 0 | 0.56 | 0.6 | 0.52 | No | Psychiatric Diagnosis |
| 3 | 0 | 0.56 | 0.6 | 0.52 | No | Psychiatric Diagnosis |
| 4.5 | 1 | 0.72 | 0.78 | 0.67 | No | Psychiatric Diagnosis |
| 0 | 0 | 1.18 | 1.29 | 1.07 | Yes | Psychiatric Diagnosis |
| 1 | 0 | 1.32 | 1.43 | 1.21 | Yes | Psychiatric Diagnosis |
| 2 | 0 | 1.39 | 1.51 | 1.28 | Yes | Psychiatric Diagnosis |
| 3 | 0 | 1.39 | 1.5 | 1.27 | Yes | Psychiatric Diagnosis |
| 4.5 | 0 | 1.21 | 1.37 | 1.06 | Yes | Psychiatric Diagnosis |
| 4.5 | 1 | 1.49 | 1.63 | 1.36 | Yes | Psychiatric Diagnosis |
